# Propofol-diazepam or propofol-midazolam co-induction in healthy dogs: effects on propofol dosages, cardiovascular and respiratory events

**DOI:** 10.18849/ve.v7i2.450

**Published:** 2022-05-04

**Authors:** Joshua Coward+, Mathieu Raillard+

**Keywords:** ANAESTHESIA, BENZODIAZEPINES, CO-INDUCTION, DIAZEPAM, DOGS, INDUCTION, MIDAZOLAM, PROPOFOL

## Abstract

PICO question

In healthy dogs, does the use of diazepam or midazolam administered in co-induction with propofol result in a reduction in the dose of propofol required to induce anaesthesia and a decrease in adverse cardiovascular and respiratory events?

Clinical bottom line

Category of research question

Treatment

The number and type of study designs reviewed

Eight papers were critically reviewed. A total of six manuscripts were prospective, randomised, blinded, clinical studies. One trial was prospective, randomised, blinded, clinical with a Latin square, incomplete design. One study was retrospective, randomised, blinded, crossover, experimental

Strength of evidence

Moderate

Outcomes reported

Variables assessed in this Knowledge Summary included: propofol dose required to induce anaesthesia (considering successful orotracheal intubation as an end point), changes in cardiovascular variables (heart rate, systolic, mean and diastolic blood pressure) and changes in respiratory variables (development of apnoea, changes in respiratory rates)

Conclusion

In healthy dogs, using propofol-diazepam or propofol-midazolam co-induction resulted in a reduction in propofol dose required to induce anaesthesia in some trials only. Midazolam appeared more effective than diazepam in this context. The dosages, timing and sequence of drug administration seemed relevant. No evidence suggested that using propofol-diazepam or propofol-midazolam co-induction resulted in a reduction of adverse cardiovascular or respiratory events. In addition, although this was out of the scope of the PICO question addressed here, adverse events (e.g. excitement, poorer quality of induction) were reported in several studies when diazepam or midazolam were used in co-induction

How to apply this evidence in practice

The application of evidence into practice should take into account multiple factors, not limited to: individual clinical expertise, patient’s circumstances and owners’ values, country, location or clinic where you work, the individual case in front of you, the availability of therapies and resources.

Knowledge Summaries are a resource to help reinforce or inform decision making. They do not override the responsibility or judgement of the practitioner to do what is best for the animal in their care.

## The evidence

Eight randomised clinical trials of moderate strength were reviewed to identify if the administration of diazepam or midazolam, used as co-induction agents with propofol, resulted in a reduced dose of propofol and in turn a reduction of adverse cardiovascular and respiratory events in healthy dogs. In spite of a number of confounding factors, there is some evidence to suggest that midazolam (more than diazepam), at certain dosages, may reduce the amount of propofol required to induce anaesthesia. Currently, there is no evidence to suggest this results in a reduction of adverse cardiovascular or respiratory events.

## Summary of the evidence

**Braun et al. (2007):** 

Population:	Recruitment: Randomly sourced research dogs of unknown gender and breed. Criteria for eligibility and inclusion: Considered ‘healthy’ on physical exam. Packed cell volume (PCV) between 35.0% and 57.0%. Total protein value between 5.2 g/dL and 7.3 g/dL. Criteria for exclusion and rejection: Dogs estimated at < 6 months or > 5 years of age. Other population information: Weights ranged from 10.5–27 kg (mean ± SD, 21.4 ± 7.5 kg). Body condition score range 2–7 out of 9 (mean ± SD, 4.8 ± 1.2).
Sample size:	25 dogs.
Intervention details:	Random allocation into intervention groups: Intravenous (IV) 0.9% saline at 0.1 ml/kg (n = 9); IV 2% lidocaine at 2 mg/kg (n = 8); IV diazepam at 0.25 mg/kg (n = 8). Administration of intervention: All subjects received a 20 G 1-inch cephalic catheter and 22 G 1-inch dorsal pedal arterial catheter then at least 20 minutes cage rest. Pre-oxygenation for ≥ 5 minutes before measurements began. Co-induction agent was injected via covered syringe. 2 minutes later IV propofol (0.8 mg/kg) was administered incrementally every 6 seconds up to a maximum of 8 mg/kg or until jaw tone allowed for intubation, assessed by the same anaesthetist for all subjects. Atracurium (0.3 mg/kg) given then intubated.
Study design:	Prospective randomised, blinded, clinical study.
Outcome studied:	Effect of co-induction agent on propofol dose required for intubation: Total dose of propofol required for observer to begin intubation, subjectively assessed by jaw tone. Effect of co-induction agent on cardiovascular variables: Systolic arterial blood pressure (SAP), diastolic arterial blood pressure (DAP), and mean arterial blood pressure (MAP) before co-induction agent, before induction, and before intubation. Heart rate (HR) before co-induction, before induction, and before intubation.
Main Findings (relevant to PICO question)	Propofol dose required for induction was lower with diazepam than saline alone but this finding was not statistically significant. Cardiovascular parameters were not statistically affected by diazepam use as a premedication at any time point.
Limitations:	Broad inclusion criteria (unknown origin, prior health status, age, and breed of dogs) which may add variability in the population studied given the relatively small sample size. Subjective nature of jaw tone analysis as indicator of anaesthetic depth. As it was assessed by an experienced anaesthetist it is a more reliable assessment, but still subjective in nature. Sample size was only calculated retrospectively and deemed sufficient. Measurements stopped prior to intubation, limiting how useful this information is in a clinical situation.

**Covey-Crump & Murison (2008):** 

Population:	Recruitment: 67 client-owned dogs scheduled to undergo anaesthesia. Criteria for eligibility and inclusion: American Society of Anesthesiologists (ASA) Score of I or II. Criteria for exclusion and rejection: Boxers and other giant breed dogs. Nervous or aggressive dogs. Significant trauma or systemic disease. Recent history of drug therapy with potential effects on sedative/anaesthetic agents. Other population information: 35 male dogs, 31 female dogs. Age 6–20 months. Weight ranged from 4.7–48.0 kg.
Sample size:	66 dogs; one dog excluded from results due to extravascular propofol administration.
Intervention details:	Random allocation into intervention groups: Control-propofol (CP) intravenous (IV) heparinised saline at 0.04 ml/kg (n = 22); Fentanyl-propofol (FP) IV fentanyl at 2 µg/kg (n = 22); Midazolam-propofol (MP) IV midazolam at 0.2 mg/kg (n = 22). Administration of intervention: All subjects received intramuscular (IM) 0.025 mg/kg acepromazine + 0.25 mg/kg morphine sulphate premedication. After 30 minutes cephalic IV access was obtained. Oxygenated via mask for 3 minutes before co-induction and during induction. Co-induction agents were administered over 30 seconds and then flushed with heparinised saline. 2 minutes later IV propofol (4 mg/kg/min) was administered until lateral recumbency with absence of head/jaw/tongue movement and jaw tone allowed for intubation. The same anaesthetist administered all interventions. Intubation occurred 30 seconds after cessation of propofol administration. Anaesthesia maintained via 1% halothane gas delivered in 33% oxygen and 66% N_2_O.
Study design:	Prospective, randomised, blinded, clinical study.
Outcome studied:	Effect of co-induction agent on propofol dose required for intubation: Total dose of propofol required for observer to begin intubation, subjectively assessed by jaw-tone and muscle movement. Effect of co-induction agent on cardiovascular and respiratory parameters: Mean arterial blood pressure (MAP) before co-induction, before induction, at intubation, 2 minutes post-intubation, and 5 minutes post-intubation. Pulse rate (PR) before co-induction, before induction, at intubation, 2 minutes post-intubation, and 5 minutes post-intubation. Respiratory rate (RR) before co-induction, before induction, at intubation, 2 and 5 minutes post-intubation. Incidence of coughing, hypotension, bradycardia, panting, and apnoea.
Main Findings (relevant to PICO question)	MAP was highest in MP but not significantly higher than other groups. Mean PR was statistically higher for MP group than other groups. RR was not significantly different between groups. No statistically significant drop in propofol dose required in MP group. Incidence of coughing, hypotension, bradycardia, panting, and apnoea not statistically different between groups. Apnoea was highest in MP.
Limitations:	Dog breeds and groupings were not entirely even but group averages were fairly consistent across groups. Timing of catheter placement and co-induction varied slightly between dogs, which could have affected excitement levels and physiological variables. Only overall incidence rates for adverse events recorded but some dogs had more than one complication which was not provided. Loss of some data from oscillometric measurement of MAP and visually monitoring of RR due to panting could have minor effects on the data. The use of a premedication (acepromazine + opioid) could have an effect on the occurrence of cardiovascular or respiratory events and their severity, though this is a clinically-relevant protocol.

**Hopkins et al. (2014):** 

Population:	Recruitment: 17 client-owned animals scheduled for elective surgery or diagnostic procedure requiring anaesthesia. Criteria for eligibility and inclusion: American Society of Anesthesiologists (ASA) Score of I or II. Criteria for exclusion and rejection: ASA Score ≥ Patient condition was contraindicated for the drug protocol. Excessively nervous or aggressive patients. Other population information: Mean weight (± SD): 28 kg (± 18 kg). Mean age (± SD): 4.9 years (± 3.9 years).
Sample size:	17 dogs.
Intervention details:	Random allocation into intervention groups: CP: Intravenous (IV) 0.9% saline at 0.04 ml/kg (n = 8); MP: IV midazolam at 0.2 mg/kg (n = 9). Administration of intervention: All subjects received IM 0.025 mg/kg acepromazine + 0.25 mg/kg morphine sulphate premedication. After 30 minutes cephalic IV access was obtained. Patients were given flow-by preoxygenation for 3–5 minutes at 4 L/min before and throughout the induction. The intervention drugs were given IV manually over 15 seconds. The same anaesthetist administered all interventions. 1% propofol CRI was given at 3 mg/kg/min via a calibrated syringe pump. Once ventro or ventromedial eye position, relaxed jaw tone, and absence of tongue movement were satisfactory, endotracheal intubation was achieved. Following intubation patients were maintained on Isoflurane and oxygen (1–3 L/min) for 5 minutes.
Study design:	Clinical, randomised, blinded, prospective study.
Outcome studied:	Effect of midazolam co-induction on propofol dose required for intubation and induction: Total dose of propofol required for intubation. Effect of premedication on cardiovascular and respiratory parameters: % Changes between baseline systolic arterial blood pressure (SAP) and SAP at intubation and 5 minutes post-intubation on isoflurane. % Changes between baseline heart rate (HR) and HR at intubation and 5 minutes post-intubation on isoflurane.
Main Findings (relevant to PICO question)	Median dose of propofol required for intubation was significantly lower for MP (2.8 mg/kg) compared to CP (3.4 mg/kg). Changes between baseline SAP and SAP not statistically different at intubation. After 5 minutes, SAP decreased by 23 ± 16% from baseline in MP, vs -7 ± 10% from baseline in CP. There was not statistical difference in the actual SAP values, only in the percentage change seen. No significant difference between groups found in HR at any time point Apnoea occurred in 4/8 MP dogs and 1/8 CP but was not statistically significant (p = 0.14).
Limitations:	The study was powered to identify differences in propofol doses but not SAP or HR values, therefore conclusions on those variables may be questionable. The amount of isoflurane was not standardised following induction and the depth of anaesthesia may have varied which could affect respiratory events seen and cause some changes in cardiovascular parameters. SAP (as opposed to mean arterial blood pressure [MAP]) and HR were the only cardiovascular parameters measured.

**Ko et al. (2006):** 

Population:	Recruitment: Eight mixed-breed hound-type dogs. Criteria for eligibility and inclusion: Not reported. Criteria for exclusion and rejection: Not reported. Other population information: Weights ranged from 18–23 kg (mean ± SD, 21.2 ± 2 kg). 1 year old. Female.
Sample size:	8 dogs.
Intervention details:	Random allocation into intervention groups: Intravenous (IV) high dose diazepam at 0.4 mg/kg; IV low dose diazepam at 0.2 mg/kg; IV ‘microdose’ medetomidine at 1 µg/kg; IV placebo (physiological saline) at 0.5 ml. Administration of intervention: Preplaced cephalic IV catheter. Co-induction agent injected. IV propofol injection started 45 seconds later (approximately 8 mg/kg per 1.5 minutes, stopped for 6 seconds after half of the propofol dose was administered) up to a maximum of 8 mg/kg or until jaw tone allowed for intubation.
Study design:	Prospective, randomised, blinded, crossover, experimental study.
Outcome studied:	Effect of co-induction agent on propofol dose required for intubation: Total dose of propofol required to allow intubation (see limitations). Effect of co-induction agent on cardiovascular and respiratory variables: Electrocardiogram (ECG) to assess the presence/absence/onset of arrhythmias. Heart rate (HR) (from ECG, confirmed through auscultation) 5 minutes prior to any drug administration, one minute after intubation and every 2 minutes thereafter. Respiratory rate (RR) (auscultation) 5 minutes prior to any drug administration and every 2 minutes after the injection of the first drug/saline, one minute after intubation and every 2 minutes thereafter. Systolic arterial blood pressure (SAP), diastolic arterial blood pressure (DAP), and mean arterial blood pressure (MAP) (oscillometry) 5 minutes prior to any drug administration, one minute after intubation and every 2 minutes thereafter.
Main Findings (relevant to PICO question)	Propofol dose required for induction was 36% lower with the high-dose diazepam than with the placebo. There was no statistically significant propofol dose reduction with the low dose diazepam. Compared to baseline, HR increased with both low and high dose diazepam, not with placebo. In the placebo group, MAP was significantly lower 1 minute after induction.
Limitations:	The study title is focusing on sedation although the end-point is intubation. No justification for the number of animals included. Real age / age range of the dogs is unclear. Limited description of the dog population and health status. No exclusion criteria. Randomisation process not described. Timeline of the sequence of events prior to induction not detailed. Blinding process of the investigator not described (different volumes of intervention drugs/placebo). Unclear end points determining the end of propofol injection: discrepancy between methods and results (methods suggesting that animals receive slowly either 4 or 8 mg/kg of propofol though results report dose differences between groups).

**Liao et al. (2017):** 

Population:	Recruitment: 10 cross-bred research hound dogs. Criteria for eligibility and inclusion: Considered ‘healthy’ on physical exam, complete blood count, and biochemistry panel. Other population information: Mean age (range): 3.4 years (1.9–5 years). Mean weight (± SD): 24.5 kg (± 3.1 kg).
Sample size:	10 dogs.
Intervention details:	**Random allocation into intervention groups:**Dogs were assigned to receive one of four interventions, and procedures were repeated every seven days with a different intervention: P-S: Intravenous (IV) propofol (10 mg/ml) at 1 mg/kg + 0.9% saline at 0.06 ml/kg (n = 9); P-M: IV propofol (10 mg/ml) at 1 mg/kg + IV midazolam (5 mg/ml) at 0.3 mg/kg (n = 9); A-S: IV alfaxalone (10 mg/ml) at 0.5 mg/kg + 0.9% saline at 0.06 ml/kg (n = 10); A-M: IV alfaxalone (10 mg/ml)+ IV midazolam (5 mg/ml) at 0.3 mg/kg (n = 9). Administration of intervention: A 20 G IV cannula was placed into a cephalic vein. IV meloxicam (0.1 mg/kg) was administered. Subjects were placed in lateral recumbency**.** IV fentanyl (50 µg/ml) was injected at a dose of 7 µg/kg. 10 minutes later, induction started with the syringes and the infusion tubes covered by drapes to keep an investigator blinded**.** Propofol (1 mg/kg) (or alfaxalone 0.5 mg) was administered as a bolus, flushed with 2 ml of saline, immediately after which midazolam or saline was administered. Additional boluses of 25% of propofol or alfaxalone were given as requested every 6 seconds until loss of palpebral reflex and jaw tone allowed for intubation as determined by a single researcher. Once intubated, propofol (at a rate of 250 µg/kg/min) or alfaxalone (at a rate of 70 µg/mg/min) was administered via syringe pump to achieve total intravenous anaesthesia (TIVA). Oxygen was provided via rebreathing circuit at a flow rate of 1.5–5 l/min. TIVA was maintained at the lightest possible plane (determined by anaesthetist) for a separate experimental procedure.
Study design:	Prospective, randomised, blinded, latin square, incomplete, clinical study.
Outcome studied:	Effect of co-induction agent on dose required for induction: Total dose of propofol required for tracheal intubation and to begin TIVA.
Main Findings (relevant to PICO question)	Propofol dose required for induction with midazolam was lower than with saline. 28.6% reduction in propofol dose.
Limitations:	Incomplete Latin Square design limited the number of patients who received all interventions and each dog received varying number of procedures (four dogs received five treatments, five received three, and one received two). There is also no breakdown provided of which dogs were allocated to each group. The percentage by which midazolam reduced the dose of induction agent may have been affected by the fentanyl premedication. Despite having 7 days rest between procedures, the repeated anaesthetic procedures could impact the total dose of propofol required for induction. The sample size was very small (calculated by authors to be a minimum of four dogs per group to detect significant changes), and some dogs received the same intervention on more than one occasion. Key parameters seen in other studies, such as body condition score, were not included in inclusion criteria which could cause considerable variability in subjects.

**Minghella et al. (2016):** 

Population:	Recruitment: 60 client-owned dogs undergoing elective surgical procedures. Criteria for eligibility and inclusion: American Society of Anesthesiologists (ASA) Score of I or II. Criteria for exclusion and rejection: Brachycephalic. Significantly overweight. Age < 6 months or > 8 years. Receiving opioids. History of vomiting, regurgitation, or respiratory obstruction. Other population information: 29 male dogs, 31 female dogs. Mean weight (± SD): 29 kg (± 9.7 kg). Mean age (± SD): 48 months (± 27 months).
Sample size:	60 dogs.
Intervention details:	Random allocation into intervention groups: SG: Intravenous (IV) 0.9% saline at 5 ml/patient (n = 20); LG: IV 2% lidocaine at 2 mg/kg (n = 20); MG: IV midazolam (5 mg/ml) at 0.2 mg/kg (n = 20). Administration of intervention: All subjects received acepromazine (2 mg/ml) 0.03 mg/kg or up to a maximum dose of 1 mg + 0.2 mg/kg morphine sulphate (10 mg/ml) IM (into the epaxial muscles of the neck). After 30 minutes, the dogs were moved to a new room, placed in sternal recumbency and a cephalic vein was catheterised. Pre-oxygenation via facemask was administered for ≥ 3 minutes at 200 ml/kg/min before induction commenced. IV propofol target-controlled infusion (TCI) was administered via syringe pump (calibrated to patient age and weight) to achieve a plasma target concentration of 1 µg/ml. After 3 minutes at the target concentration, the intervention drugs (diluted with 0.9% saline to a total volume of 5 ml) were administered IV over 30 seconds. After 2 minutes, palpebral reflex, rostro-medial rotation of eye, reduction of jaw tone and absence of tongue withdrawal reflex were assessed. If conditions were met, intubation was attempted. If not, plasma concentration target for propofol was increased by 0.5 µg/ml every 60 seconds until intubation was achievable.
Study design:	Prospective, clinical, randomised, blinded study.
Outcome studied:	Effect of co-induction agent on propofol dose required for intubation: Total dose of propofol required for observer to begin intubation, assessed by weakened palpebral reflex, rostro-medial rotation of eye, reduction of jaw tone, and absence of tongue withdrawal reflex. Effect of co-induction agent on cardiovascular parameters: Systolic arterial blood pressure (SAP), diastolic arterial blood pressure (DAP), and mean arterial blood pressure (MAP) were measured before induction via TCI (B0), and before intubation (BI), following intubation (T0), 3 minutes post-intubation (T3), and 5 minutes post-intubation (T5). Heart Rate was measured at B0, BI, T0, T3, and T5. Effect of co-induction agent on respiratory parameters: Respiratory rate (RR) was measured at B0, BI, T0, T3, and T5. End-tidal partial pressure of carbon dioxide (P_E_’CO_2_) was measured at T0, T3, and T5.
Main Findings (relevant to PICO question)	The plasma TCI range of propofol required for induction was 1.5 (1.0–4.0) µg/ml for MG, significantly lower than SG (3.0 (2.0–5.0) µg/ml). Cardiovascular and respiratory variables were not statistically affected by co-induction agent. Incidence of apnoea was not seen in any subjects nor was decreased arterial haemoglobin oxygen saturation (SpO_2_) below 98%.
Limitations:	The use of acepromazine and morphine in premedication could have affected the total dose required for induction, and the lack of occurrence of apnoea. However, this premedication is clinically relevant. The use of plasma concentration of propofol is not common in clinical practice making it difficult to directly translate these results to a clinical setting. It would have been more valuable to the readers if the authors had also reported the total dose injected alongside the plasma target concentration. It is not stated if a single observer or multiple observers were responsible for assessing readiness for intubation which could introduce variability into results.

**Robinson & Borer-Weir (2013):** 

Population:	Recruitment: 90 client-owned dogs undergoing general anaesthesia at a referral hospital for a variety of reasons. Criteria for eligibility and inclusion: American Society of Anesthesiologists (ASA) Score I–III. Deemed ‘healthy’ after pre-anaesthetic evaluation. Criteria for exclusion and rejection: Underlying condition preventing use of anaesthetic protocols. Previous sedative drug administration > 4 hours prior to procedure. Other population information: Median age (range): 4.6 (2.3–4) years. Median body mass (range): 21.5 (9.4–3) kg. Mixed signalment, with Labradors (n = 13) and cross breeds (n = 12) being the most represented.
Sample size:	90 dogs.
Intervention details:	Random allocation into intervention groups: Intravenous (IV) 0.9% Saline at 0.1 ml/kg (n = 10); IV Midazolam (5 mg/ml) at 0.2 mg/kg (n = 10); IV Midazolam (5 mg/ml) at 0.3 mg/kg (n = 10); IV Midazolam (5 mg/ml) at 0.4 mg/kg (n = 10); IV Midazolam (5 mg/ml) at 0.5 mg/kg (n = 10); IV Diazepam (5 mg/ml) at 0.2 mg/kg (n = 10); IV Diazepam (5 mg/ml) at 0.3 mg/kg (n = 10); IV Diazepam (5 mg/ml) at 0.4 mg/kg (n = 10); IV Diazepam (5 mg/ml) at 0.5 mg/kg (n = 10). Administration of intervention: IV catheter placed in the cephalic or saphenous vein. Animals were assigned to interventions via random draw and drugs were prepared by an outside qualified person before being covered by opaque tape. Premedication of 0.01 mg/kg acepromazine (2 mg/ml) and 0.2 mg/kg methadone (10 mg/ml) was given IV to all subjects. 15 minutes following premedication, after assigning a 1 to 5 sedation score, anaesthetic induction via 1% propofol at 1 mg/kg was given over 15–45 seconds via catheter and flushed with heparinised saline. While the anaesthetist in charge of propofol administration was turned away from the patient, a second person administered the treatment via rapid IV bolus. After administration of the intervention, one of two primary researchers assessed anaesthetic depth and if required administered additional propofol at 4 mg/kg/min until orotracheal intubation could be completed.
Study design:	Prospective, randomised, blinded, clinical study.
Outcome studied:	Effect of co-induction agent on propofol dose required for intubation: Total dose of propofol required for observer to begin endotracheal intubation.
Main Findings (relevant to PICO question)	Propofol dose required for induction when combined with midazolam and grouped across dose ranges was lower than that of the saline control group (p < 0.001) with a median reduction of 0.88 mg/kg (38%) and diazepam (p = 0.008) with a median reduction of 0.35 mg/kg (18%). Diazepam did not produce a significant reduction in dose requirement when compared to saline (p = 0.089). The only dose rate of midazolam which provided a significantly reduction compared to saline was 0.4 mg/kg (p = 0.014). Adverse cardiovascular and respiratory events were recorded and reported, but none proved statistically significant between any of the treatment groups.
Limitations:	Discrepancy between text and table on body weight. The definition of a healthy patient for inclusion/exclusion was not provided. ASA status between groups was statistically different with more ASA III subjects in the saline group and more ASA I subjects in the midazolam groups. Diazepam may require a longer period of time or a higher dose to see effects on propofol dose requirements than used in this study. Variation in sedation score following premedication was present and had a significant effect on the propofol dose requirement on animals which were already profoundly sedated. Body mass was also identified as having a significant impact on the overall dose required with subjects < 5 kg requiring a higher dose per kg to achieve sedation.

**Sánchez et al. (2013):** 

Population:	Recruitment: 33 client-owned dogs scheduled for surgery under general anaesthesia. Criteria for eligibility and inclusion: American Society of Anesthesiologists (ASA) Score I–III. Criteria for exclusion and rejection: Subjects which were extremely excited. Any conditions or diseases which affected mentation or behaviour. Other population information: 17 male dogs and 16 female dogs. Ages ranged from 0.5–10 years (mean 3.8 ± SD 3.1). Weights ranged from 5–30 kg (mean 15.3 ± SD 9.2).
Sample size:	33 dogs.
Intervention details:	**Random allocation into intervention groups **(the order of administration varied)**:** CP: Saline-propofol (n = 11); MP: Midazolam-propofol (n = 11); PM: Propofol-midazolam (n = 11). Dosage of interventions: 9% saline at 0.1 ml/kg at volume equivalent to midazolam. Intravenous (IV) midazolam at 0.25 mg/kg diluted 1:2 with saline. IV propofol at 1 mg/kg. Administration of intervention: All subjects were placed in a quiet room for 15 minutes before having baseline heart rate (HR), respiratory rate (RR), and systolic arterial blood pressure (SAP) recorded (mean of 3 measurements). Premedication of 0.02 mg/kg acepromazine maleate and 0.4 mg/kg morphine hydrochloride was given to all subjects IM in the biceps femoris. Animals were placed in a dimly lit, quiet cage for 30 minutes before being evaluated for level of sedation. A 20–22 G cephalic catheter was placed. Subjects then received the interventions. Group CP received IV saline over 1 minute followed 30 seconds later by IV propofol given over 30 seconds. Group MP received IV midazolam over 1 minute followed 30 seconds later by IV propofol given over 30 seconds. Group PM received IV propofol over 30 seconds followed 30 seconds later by IV midazolam given over 1 minute. After administration of the intervention, subjects were assessed for anaesthetic depth. A single anaesthetist administered all interventions. If required additional propofol boluses were administered at 0.5 mg/kg/min over 15 seconds until intubation could be completed. Following intubation, anaesthesia was maintained with isoflurane at 2.5% in 100% oxygen for 10 minutes and then to effect after that.
Study design:	Prospective, randomised, blinded, clinical study.
Outcome studied:	Effect of co-induction agent and order of administration on propofol dose required for intubation: Total dose of propofol required for observer to begin intubation, subjectively assessed by absence of voluntary movement, decreased palpebral reflex, jaw tone relaxation, and absence of coughing. Effect of co-induction agent and order of administration on cardiovascular parameters: SAP following acclimatisation, 30 minutes following premedication, and following intubation at 0, 2, 5, and 10 minutes; HR following acclimatisation, 30 minutes following premedication, and following intubation at 0, 2, 5, and 10 minutes. Effect of co-induction agent and order of administration on respiratory parameters: RR following acclimatisation, 30 minutes following premedication, and following intubation at 0, 2, 5, and 10 minutes. SpO_2_ and P_E_’CO_2_ following intubation at 0, 2, 5, and 10 minutes. Occurrence of apnoea (defined as lack of spontaneous ventilation for 30 seconds) and tachypnoea (defined as RR > 30 breaths/min).
Main Findings (relevant to PICO question)	Propofol dose required for induction was 47% lower in the MP (1.7 ± 6 mg/kg; p = 0.001) and 65% lower in the PM groups (1.1 ± 0.2 mg/kg; p = 0.001) when compared to the CP group (3.18 ± 0.56 mg/kg). In the PM group, a significantly lower dose when compared to the MP group was also required (p = 0.022). Incidence of apnoea was significantly higher in CP (p = 0.005) and MP groups (p = 0.031) when compared to the PM group. Tachypnoea occurred more often in the MP group than CP group during and after induction.
Limitations:	Delays in the time between propofol administration and intubation varied between groups due to the speed of delivery of the midazolam. The absence of a propofol-saline group is a strong limitation.

## Appraisal, application and reflection

Co-induction of anaesthesia using propofol with a benzodiazepine is common practice. Most protocols use diazepam or midazolam. The goal is a reduction in the dose of propofol required to induce anaesthesia, in the hope that this will be associated with a reduction in adverse cardiovascular (e.g. reduction in cardiac output, decrease in systemic vascular resistance) and respiratory events caused by propofol. Quality of induction may be altered by co-inductions. We expected a high variability in methods of assessment of quality of induction, precluding meaningful comparisons among studies. For this reason, we focused on the effects of diazepam or midazolam on propofol-dose requirement and cardiovascular and respiratory effects.

The eight papers reviewed here ranged in size from small prospective studies to moderately sized sample groups. While many studies did a satisfactory job investigating potential dose reductions for propofol, they were not necessarily powered to evaluate differences in cardiovascular and respiratory effects. Therefore, more studies specifically looking at whether this reduction decreased adverse events would be needed to make a conclusive link between these two outcomes.

Interestingly, overall, there was no evidence to support the hypothesis of a reduction in the incidence or magnitude of adverse cardiovascular or respiratory events. Other patient factors may be more relevant in healthy dogs.

Although clinically relevant, the use of premedication protocols in seven of the eight studies was a confounding variable. One consideration highlighted by Sánchez et al. (2013) which warrants further attention is the effect of the order of co-induction agent administration. Some propofol was administered before the benzodiazepine in three of the manuscripts reviewed here, two using midazolam, and one diazepam.

Ko et al. (2006) was the only study evaluating diazepam-propofol co-induction that reported a propofol dose reduction, when a ‘high dose’ diazepam (0.4 mg/kg) was used 45 seconds before induction with propofol. In contrast, in Braun et al. (2007) and Robinson & Borer-Weir (2013), the use of diazepam did not result in significant propofol dose reductions. There were differences between the studies. No premedication was administered in Braun et al. (2007) and diazepam was given at two different timings (two minutes before propofol in the first study, after a 1 mg/kg propofol bolus in the second). Robinson & Borer-Weir (2013) also evaluated several diazepam dosages. Overall, the changes brought by diazepam were less frequently reported than the changes caused by midazolam. The slower onset of diazepam compared to midazolam is a hypothesis advanced in Robinson & Borer-Weir (2013) to explain the differences observed. This could not be confirmed despite the different timings of administration observed here.

Midazolam administration was associated with propofol dose-reduction in five of the six papers which included it (Hopkins et al., 2014; Liao et al., 2017; Minghella et al., 2016; Robinson & Borer-Weir 2013; and Sánchez et al. 2013). Robinson & Borer-Weir (2013) was the only study to compare dose rates of midazolam. It found that, while the grouped dose rates showed a significant drop in propofol dose requirement compared to the control, when individually analysed only a dose of 0.4 mg/kg provided a significant result. One study using midazolam did not show any propofol-dose reduction (Covey-Crump & Murison, 2008). In that case, midazolan was administered at 0.2 mg/kg, two minutes before propofol. Some excitement was seen in some dogs in that study and in Hopkins et al. (2014). While outside the scope of this knowledge summary, Sánchez et al. (2013) found that more excitation occurred when midazolam was given prior to propofol. Excitement during the induction period can influence the quality of induction agent required to achieve oro-tracheal intubation.

In conclusion, in healthy dogs, using diazepam-propofol or midazolam-propofol co-induction resulted in a reduction in propofol dose required to induce anaesthesia in some trials only. Midazolam appeared more effective than diazepam in this context. The dosages, timing and sequence of drug administration seemed relevant. No evidence suggested that using diazepam-propofol or midazolam-propofol co-induction resulted in a reduction of adverse cardiovascular or respiratory events. In addition, although this was out of the scope of the PICO question addressed here, adverse events (e.g. excitement, poorer quality of induction) were reported in several studies when diazepam or midazolam were used in co-induction. The benefits of benzodiazepines-propofol co-inductions are questionable in a clinical context, when dealing with a healthy canine population.

## Methodology Section

**Search Strategy:** 

Databases searched and dates covered:	Medline via Ovid (1946–2022 Week 6) PubMed (1966–2022 Week 6) Scopus (1970–2022 Week 6) CAB Abstracts via Web of Science (1910–2022 Week 6)
Search strategy:	Medline via Ovid: ((dog or dogs or canine) and (co-induction or coinduction or induction) and propofol and (midazolam or diazepam)).af. limit 1 to (english language and yr="1946 - current") (NB: “.af” stands for “all fields”) PubMed:(((Dog OR Dogs OR Canine) AND (co-induction OR coinduction OR induction)) AND (Propofol)) AND (Midazolam OR Diazepam) Scopus:( TITLE-ABS-KEY ( dog OR dogs OR canine ) AND TITLE-ABS-KEY ( co-induction OR coinduction OR induction ) AND TITLE-ABS-KEY ( propofol ) AND TITLE-ABS-KEY ( midazolam OR diazepam ) ) AND PUBYEAR > 1970 AND PUBYEAR < 2023 AND ( LIMIT-TO ( LANGUAGE , "English" ) ) CAB Abstracts:TOPIC: (Dog OR Dogs OR Canine) AND TOPIC: (co-induction OR coinduction OR induction) AND TOPIC: (Propofol) AND TOPIC: (Midazolam OR Diazepam)Timespan: 1910–2022. Indexes: CAB Abstracts.
Dates searches performed:	09 Feb 2022

**Exclusion / Inclusion Criteria:** 

Exclusion:	Articles not available in English, single case reports, book chapters, conference proceedings, articles which did not answer the PICO question (Non-canine, different anaesthetic induction combinations, non-healthy animals) and literature reviews.
Inclusion:	Available in English, not retracted.

**Search Outcome:** 

Database	Number of results	Excluded – Not Related to PICO	Excluded – Non-Primary Research	Excluded – Non-English Publication	Excluded – Unable to Access	Total relevant papers
Medline via Ovid	57	49	0	0	0	8
PubMed	58	50	0	0	0	8
Scopus	164	154	2	0	0	8
CAB Abstracts	117	109	0	0	0	8
Total relevant papers when duplicates removed						8

## Conflict of interest

The authors declare no conflicts of interest.

The authors would like to acknowledge Dr. Merran Govendir for her comments and assistance in reviewing the paper.

## Disclaimer

Knowledge Summaries are a peer-reviewed article type which aims to answer a clinical question based on the best available current evidence. It does not override the responsibility of the practitioner. Informed decisions should be made by considering such factors as individual clinical expertise and judgement along with patient’s circumstances and owners’ values. Knowledge Summaries are a resource to help inform and any opinions expressed within the Knowledge Summaries are the author's own and do not necessarily reflect the view of the RCVS Knowledge. Authors are responsible for the accuracy of the content. While the Editor and Publisher believe that all content herein are in accord with current recommendations and practice at the time of publication, they accept no legal responsibility for any errors or omissions, and make no warranty, express or implied, with respect to material contained within. For further information please refer to our Terms of Use.
